# Synthesis of Polyaniline-Coated Graphene Oxide@SrTiO_3_ Nanocube Nanocomposites for Enhanced Removal of Carcinogenic Dyes from Aqueous Solution

**DOI:** 10.3390/polym8090305

**Published:** 2016-09-02

**Authors:** Syed Shahabuddin, Norazilawati Muhamad Sarih, Muhammad Afzal Kamboh, Hamid Rashidi Nodeh, Sharifah Mohamad

**Affiliations:** 1 Polymer Research Laboratory, Chemistry Department, Faculty of Science, University of Malaya, Kuala Lumpur 50603, Malaysia; syedshahabuddin@siswa.um.edu.my (S.S.); afzal82_kamboh@yahoo.com (M.A.K.); hamid_rashidi82@yahoo.com (H.R.N.); sharifahm@um.edu.my (S.M.); 2 Department of Chemistry, Faculty of Science, University of Tehran, Tehran 14174, Iran; 3 University of Malaya Centre for Ionic Liquids (UMCiL), University of Malaya, Kuala Lumpur 50603, Malaysia

**Keywords:** graphene oxide, polyaniline, nanocomposites, adsorbent, methylene blue, methyl orange

## Abstract

The present investigation highlights the synthesis of polyaniline (PANI)-coated graphene oxide doped with SrTiO_3_ nanocube nanocomposites through facile in situ oxidative polymerization method for the efficient removal of carcinogenic dyes, namely, the cationic dye methylene blue (MB) and the anionic dye methyl orange (MO). The presence of oxygenated functional groups comprised of hydroxyl and epoxy groups in graphene oxide (GO) and nitrogen-containing functionalities such as imine groups and amine groups in polyaniline work synergistically to impart cationic and anionic nature to the synthesised nanocomposite, whereas SrTiO_3_ nanocubes act as spacers aiding in segregation of GO sheets, thereby increasing the effective surface area of nanocomposite. The synthesised nanocomposites were characterised by field emission scanning electron microscopy (FESEM), transmission electron microscopy (TEM), thermogravimetric analysis (TGA), X-ray diffraction (XRD), and Fourier transform infrared spectroscopy (FTIR). The adsorption efficiencies of graphene oxide (GO), PANI homopolymer, and SrTiO_3_ nanocubes-doped nanocomposites were assessed by monitoring the adsorption of methylene blue and methyl orange dyes from aqueous solution. The adsorption efficiency of nanocomposites doped with SrTiO_3_ nanocubes were found to be of higher magnitude as compared with undoped nanocomposite. Moreover, the nanocomposite with 2 wt % SrTiO_3_ with respect to graphene oxide demonstrated excellent adsorption behaviour with 99% and 91% removal of MB and MO, respectively, in a very short duration of time.

## 1. Introduction

Water pollution poses a serious threat to the environment, thereby attracting much scientific attention to the removal of organic waste and toxic water pollutants from aqueous bodies [1]. The textile industry, one of the major worldwide contributors to water pollution, causes major impact on the quality of available water resources through deliberate or inadvertent release of dye effluents into water bodies. Dyes are complex organic molecules that adhere to the surface of fabrics, thereby imparting colour to them. There are more than 100,000 commercially available dyes [1] used in a wide variety of application including textiles [2], paper [3], tanning industries [4], plastics, printing, food processing [5,6,7], and so on. Approximately 10,000 tonnes of synthetic dyes are used per year by textile industries alone, discharging nearly 100 tonnes of dyes in water bodies as effluents [7]. Most of the synthetic dyes evade conventional water treatment methods, thereby accumulating in the environment due to their high degree of stability towards biodegradation, temperature, light, detergents, and soaps [8,9]. Methylene blue (MB) and methyl orange (MO), commercial dyes used for various applications such as textiles, papers, leathers, additives, laser printing, etc., are heterocyclic aromatic chemical compounds having complex chemical structures and synthetic origin, owing to which they are resistant to biodegradation and very stable to light and oxidation [10,11,12]. These dyes are highly toxic, persistent, carcinogenic, and mutagenic in nature. By virtue of their cationic/anionic as well as aromatic nature they are easily soluble in an aqueous/alcoholic medium and usually generate sulphur/nitric oxides at high temperature. As a result of the reduction process, these dyes reduce the dissolved oxygen, which modifies the properties as well as characteristics of aqueous fluids and can cause severe adverse health effects such as breathing difficulties, nausea, vomiting allergic dermatitis, skin irritation, cancer, and mutations [1]. Hence, for a safer environment, the removal of these noxious dyes from aqueous environment is essential. Up to now considerable efforts such as coagulation [13], photocatalysis [10,12], biological treatment [14], chemical oxidation [15], membrane separation [16], and adsorption [17,18] have been performed to eliminate noxious dyes from aqueous environment. Among all these techniques, adsorption continues to attract considerable attention due to its simplistic approach and numerous benefits such as greater efficiency, the capacity to remove dyes on a large scale, the ease of recovery, and the recyclability of adsorbents. Different classes of adsorbents such as activated carbon [19], polymeric materials [20], biomass [21], MWCNT [22], etc. have been employed to eliminate dyes from polluted water.

Presently, conducting polymers have been the focus of immense scientific attention at an academic and industrial level. Unique electrical and optoelectronic properties due to extended π-conjugated electron systems make conductive polymers extensively explored materials. Conducting polymers such as polythiophene, polyacetylene, polypyrrole, polyphenylene, and polyaniline have been widely studied in multidisciplinary research areas comprising environmental, electronics, electromagnetic, thermoelectric, sensors, batteries, electro-luminescence, and electromechanical applications [23,24,25,26,27,28]. Among the conducting polymer family, polyaniline (PANI), owing to its unique electrochemical properties, higher environmental stability, easy synthetic methodologies, cost-effectiveness, efficient thermal stability, and wide varieties of application, has been most intensively investigated by the scientific community [29]. However, several drawbacks such as poor solubility, poor mechanical properties, lower effective surface area, etc. restrict the use of PANI in many environmental applications [30]. In order to overcome these limitations, PANI is often polymerised in the presence of variety of other organic and inorganic materials to enhance its properties. Morphology and active surface area are two major characteristics that play a significant role in increasing the adsorption capacity of PANI-based composite materials; they can be manipulated by incorporating nanoscale materials in the matrix of the polymer. PANI-based nanocomposites have been extensively studied as adsorbent materials for the removal of dyes and other organic pollutants from waste waters and continue to be the most favoured contender for various environmental applications [31].

Graphene, a two-dimensional one-atom-thick sheet of all sp^2^-hybridized carbon, has received research interest due to its distinctive electronic, thermal, optical, mechanical, and excellent chemical tolerance capabilities, as well as its large surface-to-volume ratio [32,33,34,35]. One of the most attractive features of graphene is its large theoretical specific surface area (2630 m^2^·g^−1^), which makes it a suitable candidate for use as an adsorbent material [36]. Due to these distinctive properties, graphene is often used as an appropriate matrix for designing nanocomposites with other substances such as polymers [37], a metal–organic framework [38,39], metal nanoparticles [40], and so on. PANI nanocomposites with graphene oxide (GO) have demonstrated enhanced physical and chemical properties compared with neat PANI or graphene oxide and have been exploited in numerous applications [40,41,42,43]. Besides GO, inorganic metal oxide-based nanocomposites play a significant role in potential applications including photodegradation, waste water treatment through adsorption, photovoltaics, photochromism, etc. owing to their low cost, facile synthesis, large surface area, and physiochemical properties [44]. Various nanocomposite materials with inorganic metal oxides such as SrTiO_3_ [12], TiO_2_ [45], Fe_3_O_4_ [46], Co_3_O_4_ [10,47], etc. have been employed for the effective treatment of dye waste water. Therefore, GO and PANI, together with some metal oxide nanoparticles, can be explored for the synthesis of nanocomposite materials to address the present-day issue of water pollution.

In the present study we reported the facile synthesis of polyaniline-coated graphene oxide doped with SrTiO_3_ nancocube nanocomposites, synthesised through a simple in situ oxidative polymerisation technique for the adsorption of a cationic dye (MB) and an anionic dye (MO). GO was synthesised using modified Hummer’s method, whereas SrTiO_3_ nanocubes were synthesized using the simplistic hydrothermal technique and both were later incorporated into the polymer matrix during polymerization. Simultaneous removal of both cationic and anionic dyes by adsorbent is not easily achieved as the adsorbent must have the ability to attract both negative and positively charged particles. Here graphene oxide, due to the presence of oxygen-containing functionalities, attains a negative charge whereas polyaniline, due to the presence of nitrogen-containing functionalities (imine group –N= and amine group –N<), develops a positively-charged backbone, thereby causing intensive electrostatic attraction between the nanocomposites and dye molecules. SrTiO_3_ nanocubes act as spacers, which help in the segregation of GO sheets, thereby increasing the overall effective surface area of the nanocomposites. Therefore, GO and PANI, along with SrTiO_3_ nanocubes, act synergistically, imparting desired properties such as zwitterionic nature and enhanced surface area to the nanocomposites. Easy preparation, cheaper costs and superior adsorption capacity for anionic and cationic dyes projects these nanocomposites as the potential adsorbent material for waste water treatment.

## 2. Experimental Section 

### 2.1. Materials

Aniline (Fluka, St. Louis, MO, USA, ≥99%) was distilled under reduced pressure and stored in the dark before use. Ammonium peroxydisulfate (APS) (Merck, Kenilworth, NJ, USA, ≥99%), strontium hydroxide octahydrate (Sigma Aldrich, St. Louis, MO, USA, 95%); titanium(IV) oxide, anatase (Sigma Aldrich, St. Louis, MO, USA, 99.7%), hydrochloric acid, HCl (Merck, Kenilworth, NJ, USA, 37%), sulphuric acid, H_2_SO_4_ (Sigma Aldrich, St. Louis, MO, USA, 98%), methanol (Merck, Kenilworth, NJ, USA, 99.9%), potassium permanganate (Sigma Aldrich, St. Louis, MO, USA, ≥99%), hydrogen peroxide (Sigma Aldrich, St. Louis, MO, USA, 30%), graphite powder (Sigma Aldrich, St. Louis, MO, USA, ≥99.99%), and ammonia solution (R & M Chemicals, Essex, UK, 25%) were used as received without further purification. All of the reagents that were involved in the experiments were of analytical grade. Deionised water (DI) was used throughout the entire study.

### 2.2. Synthesis of Graphene Oxide (GO)

GO was synthesised using a modified Hummer’s method, as reported elsewhere [48]. Briefly, concentrated sulphuric acid (120 mL) was added into a three-neck round bottom flask (500 mL) immersed in ice bath under continuous stirring at 500 rpm. Five grams of graphite powder were added into ice-cooled concentrated sulphuric acid, followed by subsequent addition of 2.5 g NaNO_3_ and 15 g KMnO_4_. After a while the ice bath was removed and the reaction mixture was stirred overnight. When the colour of the reaction mixture turned light grey and converted into a paste, 150 mL of deionised water were added gradually into the mixture to dilute the paste. At this point the temperature of the reaction vessel was raised to 98 °C under continuous stirring for 2 h. Then 50 mL of H_2_O_2_ were added and the reaction mixture was stirred for 30 min. The reaction mixture was then filtered and washed with 5% HCl until the filtrate became colourless. Later it was washed with ethanol and subsequently with deionized water until the filtrate became neutral, and dried in a vacuum oven at 60 °C.

### 2.3. Synthesis of SrTiO_3_ Nanocubes

A typical hydrothermal technique was utilised for the synthesis of SrTiO_3_ nanocubes, as reported previously [12]. In summary, a calculated amount of strontium hydroxide octahydrate (1.4 g) was dissolved in 20 mL NaOH (3 M) under constant stirring. To this solution, titanium dioxide solution prepared by mixing 0.4 g of TiO_2_ in 20 mL NaOH (3 M) was added dropwise at a rate of one drop per second with vigorous stirring. After 30 min of stirring, 40 mL of the reaction mixture was transferred to a 100-mL Teflon-lined stainless steel autoclave and subjected to hydrothermal treatment at 130 °C for 72 h. The obtained precipitate of SrTiO_3_ nanocubes was then washed thoroughly with DI water several times and dried in a vacuum oven at 60 °C for 24 h.

### 2.4. Synthesis of Polyaniline (PANI)

Polyaniline was synthesised by the oxidative polymerisation of distilled aniline, which was dissolved in aqueous HCl (1 M), using ammonium persulfate (APS) as an oxidant. Aniline (0.0215 mol) was dissolved in 30 mL of an aqueous solution of HCl (1 M), and APS (0.0268 mol) was dissolved in 35 mL HCl (1 M). The oxidant solution was then added slowly to the aniline solution with continuous stirring at 25 °C. The reaction mixture was stirred continuously for two hours. The reaction mixture was then filtered and washed with HCl (0.5 M) until the filtrate became colourless and subsequently with DI water until the filtrate became neutral. The obtained polymer was dried in a vacuum oven at 60 °C overnight. The green colour of the obtained polymer indicated the formation of conductive polyaniline emeraldine salt.

### 2.5. Synthesis of PANI-Coated GO (GOPSr-0)

**Scheme 1:**
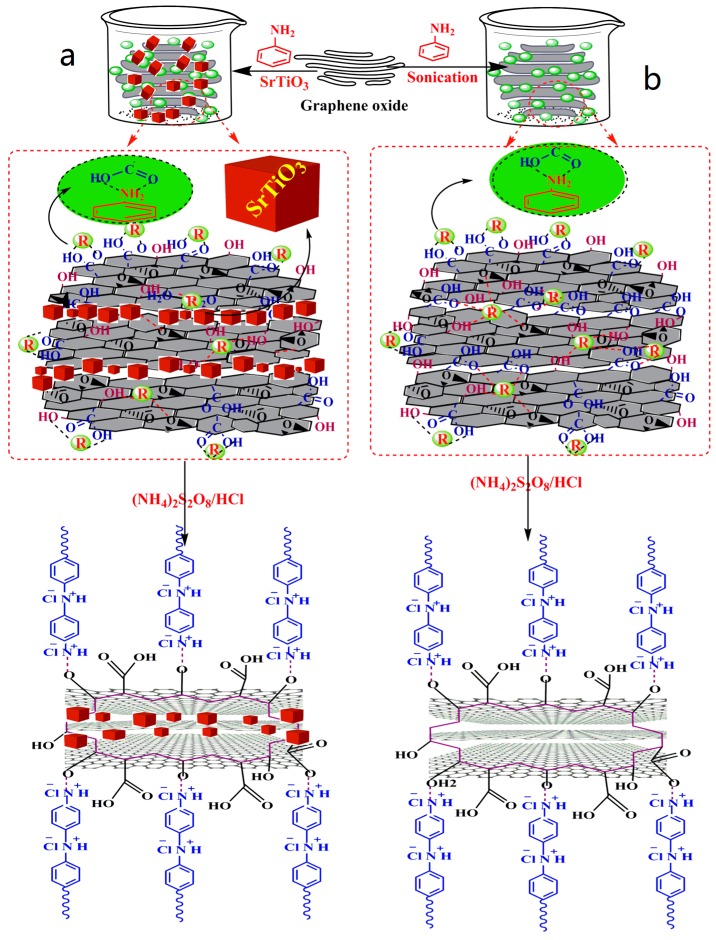
Reaction pathway for the synthesis of PANI-coated GO nanocomposite (**a**) doped with SrTiO_3_ and (**b**) without SrTiO_3_ (dotted lines represent hydrogen bonding, π–π interactions, and electrostatic interactions of PANI with GO).

PANI-coated GO nanocomposite was prepared by in situ oxidative polymerization of aniline by keeping the feed ratio of aniline to GO as 20:80. A calculated amount of aniline was dissolved in an aqueous solution of HCl (1 M). Then, a calculated amount of GO was dispersed in this solution and sonicated for 1 h. Later on, the oxidant solution (mole ratio with aniline 1:1.25) in 1M HCl was gradually added to the reaction mixture under vigorous stirring at room temperature. The reaction mixture was stirred continuously for two hours. The reaction mixture was then filtered and washed with HCl (0.5 M) until the filtrate became colourless and subsequently with DI water until the filtrate became neutral. The obtained composite was dried in a vacuum oven at 60 °C overnight and labelled as GOPSr-0. Scheme 1 represents the reaction pathway for the synthesis of GOPSr-0.

### 2.6. Synthesis of SrTiO_3_ Nanocubes-Doped, PANI-Coated GO (GOPSr)

SrTiO_3_ nanocube-doped, PANI-coated GO nanocomposites were prepared with different wt % of SrTiO_3_ (1, 2, and 5 wt % with respect to GO). A calculated amount of SrTiO_3_ nanocubes was dispersed in 5 mL of deionised water by sonication and added dropwise to an aniline–GO suspension in HCl (as described in previous section) under vigorous stirring. The resulting mixture was sonicated for 1 h until it became uniform. The work-up procedure was the same as described in the previous section. The obtained nanocomposites were labelled as GOPSr-1, GOPSr-2, and GOPSr-5 indicating 1, 2, and 5 wt % of SrTiO3 nanocubes with respect to GO, respectively. Scheme 1 represents the graphical illustration of the reaction pathway for the synthesis of SrTiO_3_ nanocube-doped, PANI-coated GO nanocomposites. 

### 2.7. Characterisation Techniques

The surface morphological and elemental analysis of the synthesised product was conducted using a JSM-7600F field emission scanning electron microscope (JEOL Ltd., Tokyo, Japan) operated at 10 kV. The size and shape of the obtained SrTiO_3_ nanocubes were studied using a JEM-2100F high-resolution transmission electron microscope (JEOL Ltd., Tokyo, Japan). X-ray diffraction (XRD) patterns were recorded using an Empyrean X-ray diffractometer (PAN analytical, Almelo, The Netherlands) from 2θ = 10° to 90° using Cu Kα radiations (λ = 1.5418 Å) at a scan rate of 0.02 s^−1^. Fourier transform infrared (FT-IR) spectra of the powdered samples were recorded using a Perkin Elmer RX1 FT-IR ATR spectrometer (Perkin Elmer, Waltham, MA, USA) in the range of 400–4000 cm^−1^ in spectral-grade KBr pellets.

### 2.8. Dye Adsorption Study

The adsorption studies were carried out using MB and MO as the model dyes in the water phase to investigate the adsorption ability of synthesised nanocomposites. The dye adsorption experiments were conducted at room temperature in a set of Erlenmeyer flasks by batch process to study the outcome of different parameters such as type of adsorbent (SrTiO_3_ nanocubes, GO, PANI, GOPSr-0, GOPSr-1, GOPSr-2, and GOPSr-5), time (0–60 min), initial pH (4.5–9.5), and effect of NaCl concentration (10–50 g/L). In general, 50 mg of the dye was dissolved in 1 L of distilled water and the solution pH was adjusted by adding HCl (0.1 N) or NaOH (0.1 N). A calculated amount of adsorbent was added to 100 mL of dye solution and kept on a shaker with constant shaking at 180 rpm. Then, 3 mL of the dye suspension were withdrawn at a regular time interval and centrifuged. The UV-visible absorption spectra of the supernatant solution were analysed using a UV-visible spectrometer (Thermo Scientific Evolution, Thermo Fisher Scientific, Waltham, MA, USA) in 1-cm quartz cuvettes to monitor the characteristic absorption peak of MB and MO. The percentage dye removal from the aqueous solution was determined according to the following equation:

(1) %*R* = {(*C*_0_ − *C*_t_)/*C*_0_} × 100,

where *C*_0_ is the initial concentration of the MO (mg/L) and *C*_t_ is the concentration of MO (mg/L) at time *t*.

## 3. Results and Discussion

### 3.1. Synthesis

**Scheme 2:**
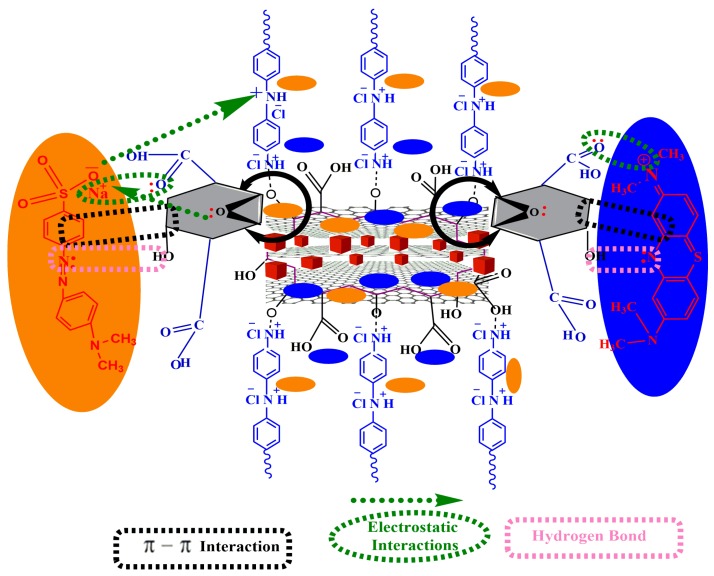
Proposed mechanism for the adsorption of MB and MO on the surface of GOPSr-2.

The main goal of this study was to design a new highly segregated, PANI-coated GO nanocomposite doped with SrTiO_3_ nanocubes, bearing cationic as well as anionic functional sites for the enhanced removal of MB and MO dyes. To achieve the desired goal, an aniline monomer containing amine functionality was introduced on the surface of GO ,which was subsequently polymerized in the presence of ammonium persulphate in acidic medium in order to obtain a PANI-coated GO nanocomposite, as shown in Scheme 1. PANI-coated GO nanocomposites contain a positively charged polymeric backbone and sp^2^ hybridized framework along with anionic functionalities. However, due to stacking of GO sheets, PANI-coated GO nanocomposite possess a substantial amount of agglomeration, which reduces the effective surface area of the adsorbent. In order to enhanced the effective surface area of SrTiO_3_, nanocubes were incorporated into the matrix of PANI-coated GO nanocomposite, where they act as spacer molecules to segregate the GO sheets, thereby increasing the total effective surface area, a prime prerequisite for an efficient adsorbent (Scheme 2).

### 3.2. Morphological Analysis of Nanocomposites 

**Figure 1:**
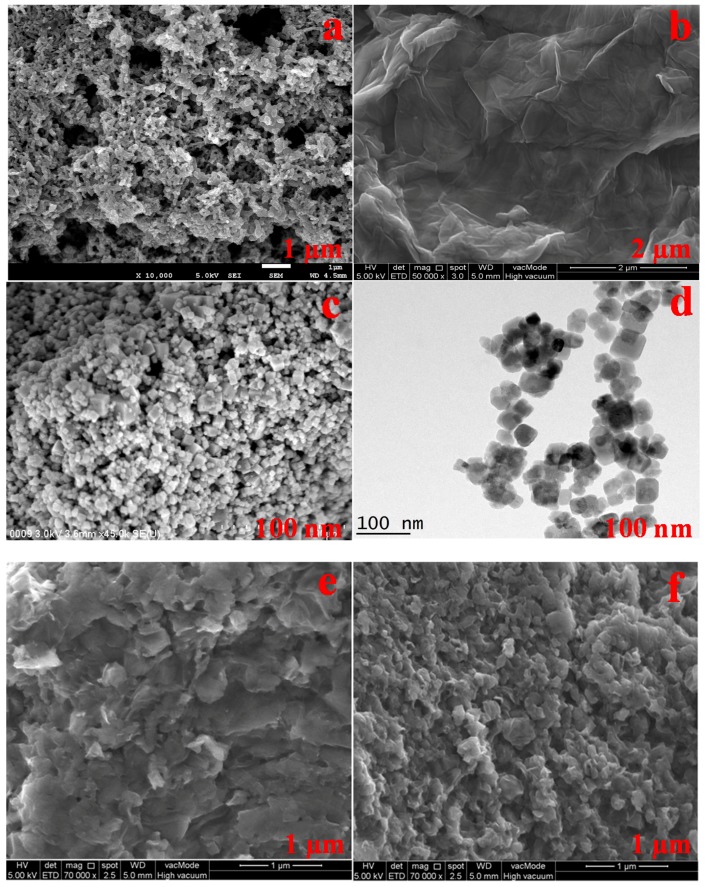
FESEM micrographs of (**a**) PANI; (**b**) GO; (**c**) SrTiO_3_ nanocubes; (**e**) GOPSr-0; and (**f**) GOPSr-2 nanocomposite; and (**d**) TEM image of SrTiO_3_ nanocubes.

The morphology and structure analysis of the synthesised graphene oxide, PANI, SrTiO_3_ nanocubes and GOPSr-2 nanocomposite were studied through FESEM. Figure 1a explicates the morphology of PANI, which displayed a specific flake-like structure. Figure 1b reveals the surface morphology of GO, clearly illustrating the disorderly stacked, folded sheet-like accumulation. Figure 1c depicts the morphology of an SrTiO_3_ nanocube. As evident from the micrographs, SrTiO_3_ nanoparticles have attained cube-shaped morphology with approximately uniform particle size. Moreover, to confirm the formation of SrTiO_3_ nanocubes, the synthesised nanoparticles were analysed via TEM. Figure 1d represents the TEM image of SrTiO_3_ nanocubes, which visibly reveals the formation of cubic particles in nanoscale, thereby confirming the formation of SrTiO_3_ nanocubes. Figure 1e exhibits PANI-coated GO nanocomposite (GOPSr-0), which indicate the transformation in the surface morphology of GO upon coating by PANI. As is obvious from the figure, the folded and stacked GO sheets become segregated after being coated by PANI. This segregation was further enhanced by doping PANI-coated GO with SrTiO_3_, as illustrated by Figure 1f. The cubic shaped SrTiO_3_ nanoparticles can be seen on the surface of the nanocomposites embedded within the layers of GO and PANI, as demonstrated by Figures S1a and b. Figure S2 exhibits the TEM images of GOPSr-2 at different magnifications. As evident from the TEM micrographs, folded sheets of GO are coated by PANI, whereas SrTiO_3_ nanoparticles can be seen embedded within the GO layers. Therefore, coating of GO with PANI and doping with SrTiO_3_ nanocubes had completely transformed the surface morphology with much segregated composite material, thereby leading to the higher surface area, a prime prerequisite for an ideal adsorbent material.

**Figure 2:**
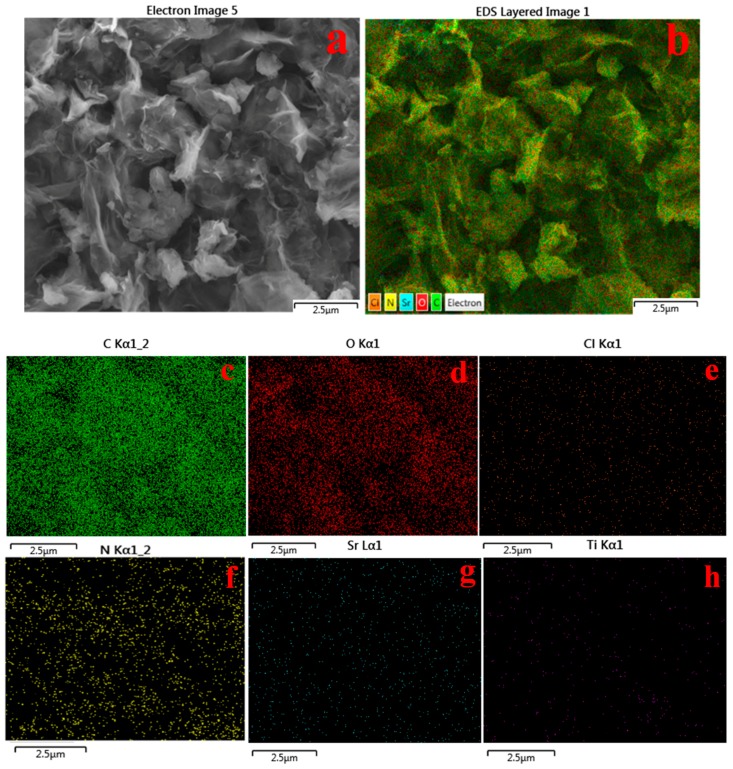
(**a**) FESEM image and (**b**) EDX elemental mapping of GOPSr-2 nanocomposite on a Si wafer for the following elements: (**c**) C; (**d**) O; (**e**) Cl; (**f**) N; (**g**) Sr; and (**h**) Ti.

Possibly because of the lower concentration of SrTiO_3_ nanocubes in nanocomposites (2 wt % with respect to GO), it is hard to observe SrTiO_3_ nanoparticles uniformly in the FESEM images of nanocomposites as these particles are embedded within the sheets of PANI-coated GO. Hence, FESEM-mapping and FESEM-EDX might perhaps be an appropriate method to validate the occurrence of SrTiO_3_ nanoparticles in the matrix of a PANI-coated GO nanocomposite. Figure 2 demonstrates the elemental mapping analysis of GOPSr-2, which reveals that SrTiO_3_ nanocubes are uniformly present within the matrix of the composite material along with carbon, oxygen, nitrogen, and chlorine. An elemental analysis (Figure S3) displays the occurrence of strontium and titanium, which additionally confirms the formation of SrTiO_3_ nanocube-doped PANI-coated GO nanocomposites.

### 3.3. FTIR Analysis

**Figure 3:**
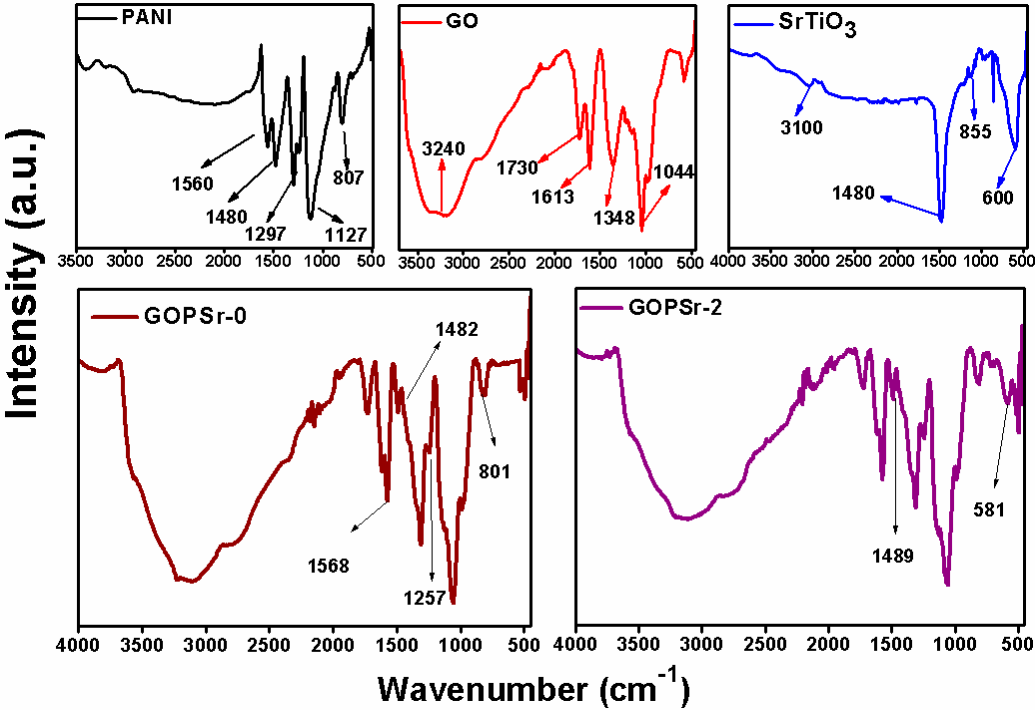
FTIR spectra of PANI, GO, SrTiO_3_, GOPSr-0, and GOPSr-2.

Figure 3 shows the FTIR spectra of PANI, GO, SrTiO_3_, GOPSr-0, and GOPSr-2. The IR bands appearing at 1560 and 1480 cm^−1^ signify the typical C–C stretching of quinoid and benzenoid rings in PANI, respectively. The peak at 1297 cm^−1^ might be ascribed to C–N and C=N stretching modes in PANI. IR peaks at 807 and 1127 cm^−1^ were attributed to out-of-plane C–H bending and in-plane C–H bending [12,49]. The IR spectrum of GO reveals a broad peak around 3240 cm^−1^, which may be attributed to the O–H stretching vibrations. The IR peaks appearing at 1730, 1613, 1395, and 1219 cm^−1^ correspond to the C=O stretching mode, sp^2^-hybridized C=C stretching and O–H bending modes, C–OH stretching mode, and C–O–C stretching mode, respectively [50]. Additionally, the peak at 1044 cm^−1^ could be attributed to the C–O vibration due to the epoxy or alkoxy groups [51]. The IR spectrum of SrTiO_3_ exhibits a band around 3100 cm^−1^, which may be attributed to the O–H stretching modes in water of crystallization. The IR peaks around 1480 cm^−1^ could be attributed to the carboxylate group stretching modes, whereas the peaks at 855 and 600 cm^−1^ are attributed to TiO_6_ octahedron bending and stretching vibration [12,52] The FTIR spectrum of PANI-coated-GO exhibits the characteristic GO band along with PANI peaks. The PANI peaks appear to be slightly shifted to 1568, 1482, 1257, and 801 cm^−1^, as revealed by the IR spectrum of PANI homopolymer. This slight shifting of peaks may be attributed to the change in chemical environment of PANI upon coating over GO, indicating some chemical interaction between PANI chains and GO matrix. The IR spectrum of an SrTiO_3_-doped nanocomposite, GOPSr-2, exhibits characteristic peaks of both GO and PANI. The band at 1489 cm^−1^ represents the SrTiO_3_ carboxylate group stretching mode, which overlaps with C–C stretching of quinoid and benzenoid and is slightly shifted. The new peak also appears at 581 cm^−1^, slightly shifted towards red from its original position (600 cm^−1^), which might be attributed to TiO_6_ octahedron bending and the stretching vibration mode. This slight shifting of the band towards red may perhaps be ascribed to some amount of weak van der Waals attraction between the SrTiO_3_ nanocubes with GO and PANI chains. Hence, the FTIR studies are in good agreement with the reported literature and evidently stipulate the formation of PANI, GO, SrTiO_3_, and SrTiO_3_-doped PANI-coated GO nanocomposites.

### 3.4. XRD Analysis

**Figure 4:**
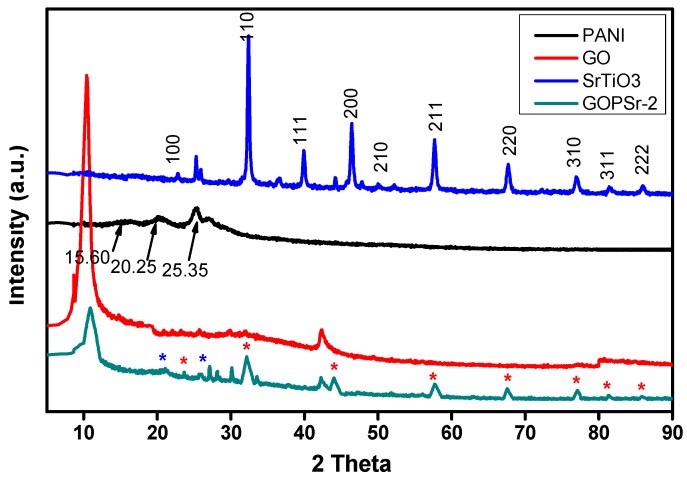
XRD pattern of PANI, GO, SrTiO3, and GOPSr-2.

Figure 4 depicts the wide-angle X-ray diffraction (WAXD) pattern of the powder samples of hydrothermally synthesized SrTiO_3_ nanocubes, GO, PANI homopolymer, and GOPSr-2. SrTiO_3_ nanocubes indicated good crystallinity with diffraction peaks corresponding to the (100), (110), (111), (200), (210), (211), (220), (310), (311), and (222) planes of cubic perovskite SrTiO_3_ structure respectively. These peaks are characteristic of SrTiO_3_ and can be readily indexed as those of cubic perovskite structure (space group: Pm3m) of SrTiO_3_ in accordance with JCPDS card No. 35-0734 [12]. The presence of well-defined and very sharp peaks in the XRD pattern of SrTiO_3_ nanocubes indicates the well-developed crystalline structure. The XRD pattern of the GO illustrated an intense and sharp peak centered at 2θ = 10.40, which corresponds to the interplanar spacing of GO sheets. The observed 2θ for GO could be attributed to the (001) reflection plane, which is usually governed by the process of synthesis and number of layers of water within the interplanar space of GO [34,42]. As is evident from Figure 4, the PANI homopolymer exhibited typical diffraction peaks at 2θ values of 15.60, 20.25, and 25.35, which are the characteristic peaks of conductive PANI. These peaks indicate the polycrystalline nature of the PANI homopolymer [53]. The peaks appearing at angles of 2θ value of 20.77 and 25.27 epitomise the periodic repetition of benzenoid and quinoid rings in PANI chains [54]. As is apparent from the XRD pattern of GOPSr-2, the intense peak of GO has significantly reduced, indicating that the aggregation of GO sheets had been considerably diminished and was abundantly utilized as the substrate by the PANI homopolymer to produce a nanocomposite hybrid material [42]. Characteristic PANI and SrTiO_3_ diffraction peaks can be seen clearly in the XRD spectrum of GOPSr-2 nanocomposites (marked by asterisks), which prove the uniform occurrence of PANI and SrTiO_3_ nanocubes in nanocomposites. Therefore, XRD investigation established the successful synthesis of SrTiO_3_, GO, and PANI and the formation of SrTiO_3_-doped, PANI-coated GO nanocomposite.

### 3.5. Adsorption Analysis of MB and MO

**Figure 5:**
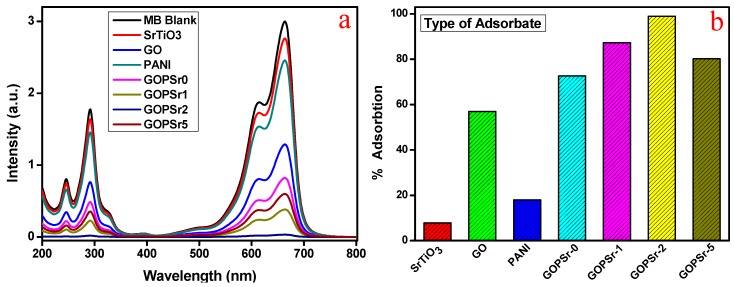
(**a**) UV-vis absorption spectra of MB aqueous in the presence of various adsorbents and (**b**) percentage removal of MB in the presence of various adsorbents (initial MB concentration: 20 mg·L^−1^; amount of adsorbent: 0.5 mg·mL^−1^; pH 7; time: 30 min; at room temperature).

**Figure 6:**
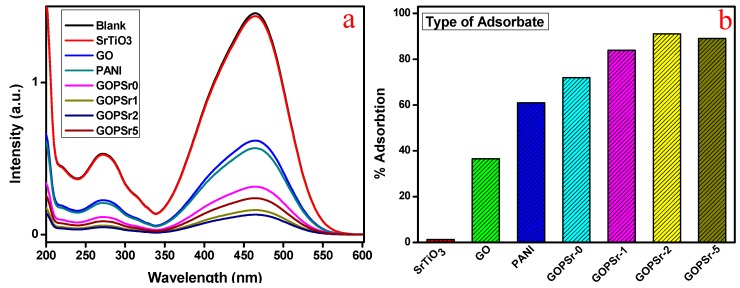
(**a**) UV-vis absorption spectra of MO aqueous in the presence of various adsorbents and (**b**) percentage removal of MB in the presence of various adsorbents (initial MO concentration: 20 mg·L^−1^; amount of adsorbent: 0.5 mg·mL^−1^; pH 7; time: 30 min; at room temperature).

The aqueous-phase adsorption behaviour of MB and MO dyes was examined in a set of Erlenmeyer flasks by batch process, using a shaker with constant shaking of 180 rpm at ambient temperature, in the presence of SrTiO_3_ nanocubes, GO, PANI homopolymer, GOPSr-0, GOPSr-1, GOPSr-2, and GOPSr-5 nanocomposites. Figure 5 and Figure 6 show the adsorption behaviour and percentage adsorption of MB and MO in the presence of various adsorbents, clearly revealing the efficient adsorption phenomenon taking place between the dyes and adsorbent molecules. The adsorption data demonstrate that SrTiO_3_ nanocubes, GO, and PANI homopolymers showed lower adsorption efficiencies as compared to the adsorption efficiencies of SrTiO_3_ nanocubes-doped, polyaniline-coated GO nanocomposites, namely, GOPSr-1, GOPSr-2, and GOPSr-5. As is apparent from Figure 5b and Figure 6b, the percentage adsorption for MB depicts the following trend: GOPSr-2 > GOPSr-1 > GOPSr-5 > GOPSr-0 > GO > PANI > SrTiO_3_, whereas the percentage adsorption for MO illustrates the following trend: GOPSr-2 > GOPSr-5 > GOPSr-1 > GOPSr-0 > PANI > GO > SrTiO_3_. Figure 5a and Figure 6a exhibit the UV-vis adsorption spectra of the MB and MO in the presence of different nanocomposites, which indicates that the adsorption efficiency of nanocomposites is greatly enhanced in the presence of SrTiO_3_ nanocubes as compared to bare GO, PANI, and PANI-coated GO, thereby predicting a synergistic phenomenon between SrTiO_3_ nanocubes, GO, and PANI. Approximately 99% of MB and 91% of MO were removed within a short duration of 30 min, demonstrating the enhanced adsorption efficiency of the GOPSr-2 nanocomposite over SrTiO_3_ nanocubes, GO, PANI homopolymer, GOPSr-0, GOPSr-1, and GOPSr-5, which exhibited nearly 8%, 57%, 18%, 78%, 87%, and 84% adsorption for MB but 1.3%, 36%, 61%, 72%, 84% and 89% adsorption for MO, respectively. Therefore, the adsorption analysis of MB and MO dyes suggests GOPSr-2 nanocomposite is an optimal adsorbent for the efficient removal of carcinogenic MB and MO dyes from aqueous solutions.

Accumulation of a substance between the liquid–solid interface or gas–solid interface due to physical or chemical associations is termed an adsorption process. With few exceptions, adsorption is usually controlled by physical parameters on most of the adsorbents such as polarity, van der Waals forces, hydrogen bonding, dipole–dipole interaction, π–π interaction, etc. [49]. Therefore, the design of an adsorbent usually depends on the type of substance to be adsorbed or removed. MB is a cationic dye that can be removed by an adsorbent showing strong affinity towards positively-charged species, whereas MO is an anionic dye that requires positively polar material for its efficient removal. GO, due to the presence of an sp^2^ hybridized framework and oxygen-containing functionalities such as hydroxyl and epoxy groups, tends to show enhanced affinity towards cationic species. As is evident from percentage adsorption data, GO alone can adsorb 57% of MB dye due to its cationic nature, whereas it only removed 36% of MO, which may perhaps be due to the formation of hydrogen bonding or van der Waal’s attractions between MO and GO. On the other hand, polyaniline in its conductive emeraldine salt state possesses a large number of amine (–N<) and imine (–N=) functional groups and substantial amounts of positive charges localised over its backbone, making it a suitable candidate for the efficient adsorption of negatively polarised substances. Thus, when PANI alone was used as an adsorbent, it was capable of adsorbing 61% of MO, while it only adsorbs 18% of MB. Thus, designing a nanocomposite material comprised of GO coated with conductive chains of PANI may be an alternative material that can adsorb MB as well as MO simultaneously. As illustrated by the percentage adsorption results in Figure 5b and Figure 6b, PANI-coated GO (GOPSr-0) efficiently adsorbed MB and MO with 78% and 72% adsorption efficiency, respectively. However, although the efficiency of PANI-coated GO has enhanced substantially, the overall performance of the nanocomposite was on the lower side. This might be due to agglomeration of disorderly stacked GO sheets, which reduces the effective surface area of the adsorbent and does not allow for efficient adsorption of dye molecules on the surface of the adsorbent. Therefore, in order to segregate the stacked GO layers, SrTiO_3_ nanocubes were incorporated inside the nanocomposites during the polymerization process (Scheme 1). These SrTiO_3_ nanocubes adhered to the sheets of GO through Sr^3+^ ions’ interactions with an sp^2^ hybridized framework of GO. Thus they act as spacers that help in the segregation of GO sheets, thereby enhancing the total effective surface area and increasing the thermal stability of the nanocomposite material, as discussed in the thermal analysis section. Hence, as revealed by adsorption results, SrTiO_3_ nanocube-doped, polyaniline-coated GO nanocomposites showed highly enhanced adsorption capacity as compared to the neat GO, PANI, and GOPSr-0. The adsorption efficiency increased to 99% for MB and 91% for MO upon doping with 2 wt % of SrTiO_3_ with respect to GO. The amount of doping percentage was optimised by varying the concentration of SrTiO_3_ and it was found that when the percentage of SrTiO_3_ increased to 5% there was a slight decrease in adsorption efficiency, which might be due to the agglomeration of SrTiO_3_ nanocubes at a high doping percentage, implying that GOPSr-2 would be an optimal adsorbent composition.

### 3.6. Effect of Adsorbent Dosage

**Figure 7:**
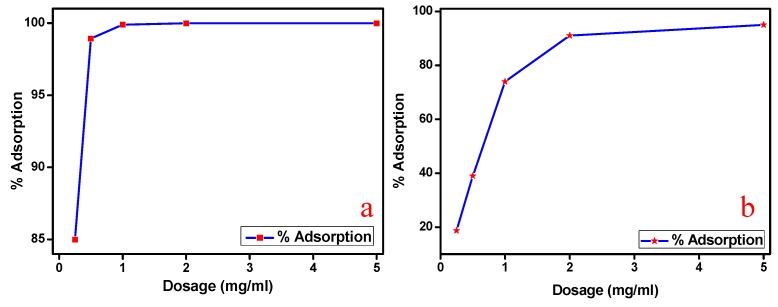
Effect of adsorbent dosage on percentage removal of (**a**) MB and (**b**) MO in the presence of GOPSr-2 (initial MB and MO concentration: 20 mg·L^−1^; pH 7; time: 30 min at room temperature).

The effect of adsorbent dosage on percentage removal of MB and MO was examined by taking different quantities of GOPSr-2 nanocomposite ranging from 0.25 to 5 mg·mL^−1^ and investigating the dye adsorption efficiency with an initial concentration of 20 mg·L^−1^, pH 7, and ambient temperature for both dyes. Figure 7 depicts the percentage adsorption of MB and MO by GOPSr-2. As is evident from the figure, the percentage adsorption of MB increases with an increase in the amount of adsorbent from 0.25 to 0.5 mg·mL^−1^ and becomes constant at a higher dosage. Approximately 85% of MB was adsorbed on the surface of the adsorbent when the dosage was 0.25 mg·mL^−1^ and increased to 99% on increasing the amount of adsorbent to 0.5 mg·mL^−1^. On the other hand, for MO, percentage removal increases gradually with the increase in adsorbent dosage from 0.25 to 0.5 mg·mL^−1^ with 19%, 39%, 74%, 91%, and 95% adsorption at an adsorbent dosage of 0.25, 0.5, 1, 2, and 5 mg·mL^−1^, respectively. The increase in adsorption efficiency when increasing the amount of adsorbent could be ascribed to the availability of more adsorption sites, and increases in the available effective surface area increased the adsorbent dosage. Moreover, the results reveal that GOPSr-2 has strong affinity toward MB as compared to MO as percentage removal was found to be 99% for MB and 39% for MO at an adsorbent dosage of 0.5 mg·mL^−1^. Therefore, 0.5 and 2 mg·mL^−1^ were selected as the optimum dosage for MB and MO, respectively, for subsequent experiments.

### 3.7. Effect of Contact Time

**Figure 8:**
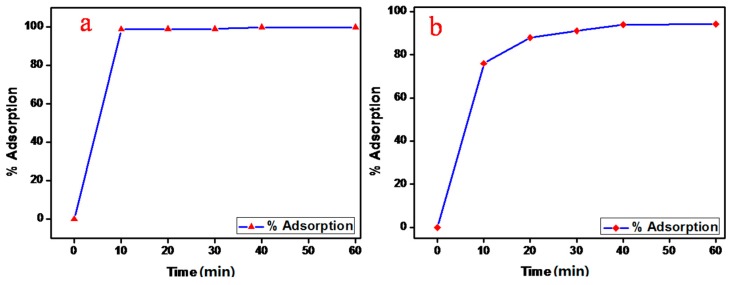
Effect of time on percentage removal of (**a**) MB and (**b**) MO in the presence of GOPSr-2 (initial MB and MO concentration: 20 mg·L^−1^; amount of adsorbent (MB): 0.5 mg·mL^−1^, (MO): 2 mg·mL^−1^; pH (MB): 9.5, (MO): 7; at room temperature).

Contact time is an important parameter to determine the efficiency of an adsorbent as a rapid rate of adsorption indicates an efficient adsorbent. In order to determine the effect of contact time on the percentage removal of dyes, adsorption was monitored at particular intervals of time ranging from 10 to 60 min with an initial concentration of 20 mg·L^−1^, pH 7, and ambient temperature using an adsorbent dosage of 0.5 mg·mL^−1^ for MB and 2 mg·mL^−1^ for MO. Figure 8 illustrates the effect of contact time on percentage removal of MB and MO. As is apparent from the figure, approximately 99% of MB was adsorbed on the surface of GOPSr-2 within 10 min, indicating rapid and efficient adsorption. Alternatively, the adsorption of MO increases steadily with the increase in time and reaches equilibrium at 40 min of contact time, with approximately 94% of dye adsorption. Thus, based upon the following experiment, 10 and 40 min were selected as the optimized contact times for further experiments.

### 3.8. Effect of pH

**Figure 9:**
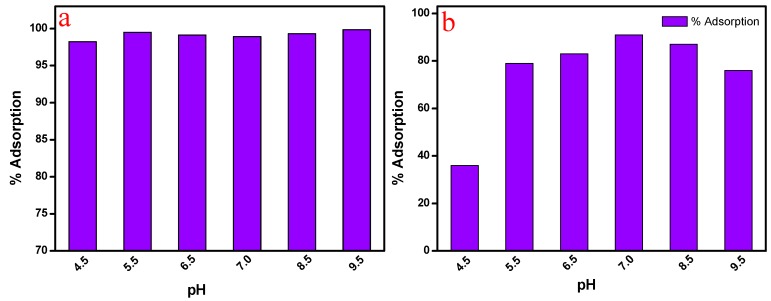
Effect of pH on percentage removal of (**a**) MB and (**b**) MO in the presence of GOPSr-2 (initial MB and MO concentration: 20 mg·L^−1^; amount of adsorbent (MB): 0.5mg·mL^−1^, (MO): 2 mg·mL^−1^; time: 30 min at room temperature).

Amongst the various physical parameters, the most significant factor influencing the efficiency of an adsorbent in wastewater treatment is the pH of the solution. The effectiveness of adsorption is reliant on the pH of the medium, since diversity in pH prompts variations in the surface properties of the adsorbent and in the degree of ionization of the dye molecules [7]. Hence, the effect of solution pH on the percentage removal of MB and MO dyes was investigated using GOPSr-2 as an adsorbent at a pH range from 4.5 to 9.5, adjusted by the addition of 0.1 N HCl or 0.1 N NaOH at ambient temperature. As depicted by Figure 9, the adsorption of MB does not show any significant change from pH 4.5 until 9.5, thereby indicating that GOPSr-2 is an excellent adsorbent material that works at a wide range of pH values. However, MO adsorption on GOPSr-2 seems to be pH-dependent, with maximum adsorption attained at pH 7. This might be explained on the basis of a p*K*a value of MO that is around 3.4. Therefore, if the p*K*a is more than the pH of MO, which predominantly exists in anionic form, it gets protonated. Additionally, in an acidic environment PANI tends to develop a positive charge on its conductive backbone due to the presence of basic (imine and amine) groups. Thus, at acidic pH values a significant amount of electrostatic repulsion occurred between the positively-charged PANI backbone and protonated MO molecules, which tends to decrease the adsorption efficiency of GOPSr-2 for MO adsorption [55]. At pH values above 7, adsorption behaviour illustrates a gradual decrease up to pH 9.5, which might be owing to the competitive adsorption of hydroxyl ions on imine and amine groups, resulting in a decline in MO adsorption. Thus, for both dyes, the maximum adsorption was achieved at a neutral pH (7), with approximately 98% adsorption for MB and 91% adsorption for MO.

### 3.9. Effect of NaCl Concentration

**Figure 10:**
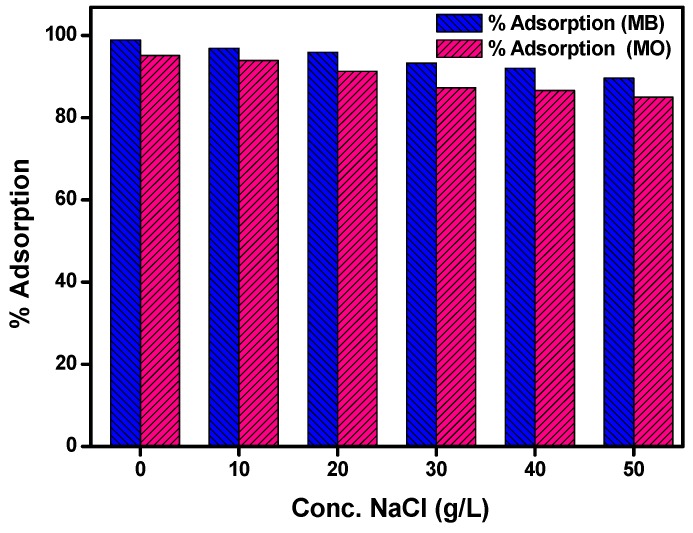
Effect of NaCl conc. on percentage removal of MB and MO in the presence of GOPSr-2 (initial MB and MO concentration: 20 mg·L^−1^; amount of adsorbent (MB): 0.5 mg·mL^−1^, (MO): 2 mg·mL^−1^; pH (MB): 9.5, (MO): 7; at room temperature).

One of the most significant features of adsorption investigations, reported by many researchers, is the effect of salt concentration on the percentage removal of dyes from industrial dye waste water. Since commercial dye waste water generally contains high salt contents, it becomes crucial to study the effect of salt concentration on the percentage removal of MB and MO from aqueous solutions using GOPSr-2 nanocomposites. Figure 10 illustrates the effect of NaCl with variable concentrations ranging from 10 to 50 g·L^−1^ on MB and MO removal percentage by GOPSr-2. As is apparent from Figure 10, the adsorption efficacy of nanocomposite is considerably influenced by the presence of NaCl. The adsorption capability of the nanocomposite declines with the increase in NaCl concentration from 10 to 50 g·L^−1^. The percentage removal decreases from 99% to 89.60% and 95% to 81.75% with an increase in concentration of NaCl of 50 g·L^−1^ for MB and MO, respectively. This reduction in adsorption efficiency may be attributed to the neutralization of the surface charge of the adsorbent by electrolyte ions that compete with dye molecules for adsorption on the surface of the nanocomposite. Nevertheless, this decline in adsorption efficiency is not very high, which implies that GOPSr-2 can effectively remove MB and MO from an aqueous solution even in the presence of a high concentration of salt.

### 3.10. Reusability Studies

**Figure 11:**
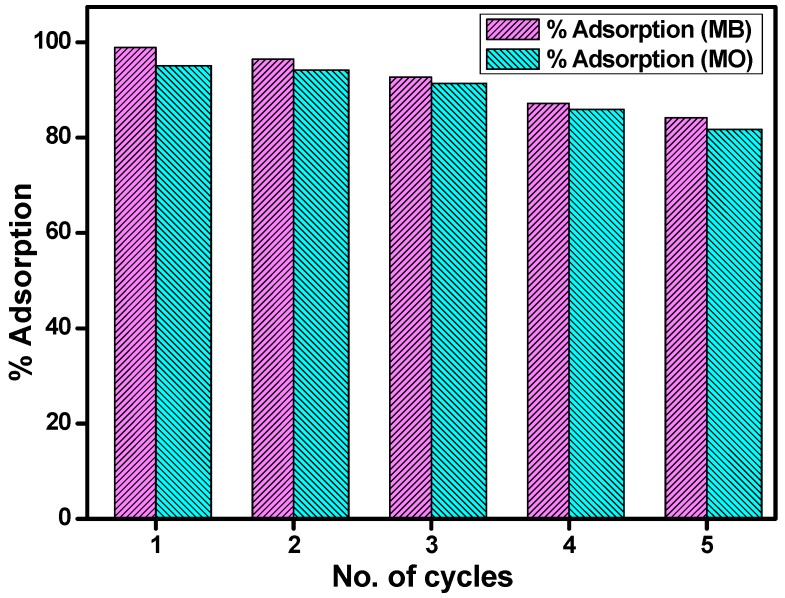
Reproducibility cycles of GOPSr-2 for MB and MO adsorption (initial MB and MO concentration: 20 mg·L^−1^; amount of adsorbent (MB): 0.5 mg·mL^−1^, (MO): 2 mg·mL^−1^; pH (MB): 9.5, (MO): 7; at room temperature).

The stability and reusability of the adsorbent are considered to be important factors for practical application and need to be thoroughly examined. Hence, reusability investigations of GOPSr-2 were conducted to examine the effect of nanocomposites on adsorption capacity after repeated usage cycles. Figure 11 illustrates the reusability of GOPSr-2 for the adsorption of MB and MO up to five cycles. After the first cycle, the adsorbent was recovered via centrifugation and filtration, followed by thorough washing with deionised water, drying at 80 °C in a vacuum oven for two hours, and successively employing it as an adsorbent for additional cycles so as to study their adsorptive efficiencies. As is apparent from Figure 11, there is a marginal decrease in the adsorption efficiency of the adsorbent after each repeated cycle. The percentage removal for MB was found to be 96.50%, 92.75%, 87.20%, and 84.15% and for MO was found to be 94.20%, 91.35%, 85.90%, and 81.75% for the second, third, fourth, and fifth cycles, respectively. This decrease in efficiency could be due to the fact that substantial unavoidable weight loss occurred during the recovery and purification of the adsorbent, which contributes to a decrease in adsorption efficiency in each repeated cycle as well as a reduction in active available sites due to some adsorbed dye molecules. Figure S4 represents the FESEM image of GOPSr-2 after the fifth reusability cycle. As is evident from Figure S4, there is no apparent change in the morphology of the nanocomposite, which indicates the high stability of GOPSr-2 even after repeated usage. However, the investigation clearly reveals that approximately 84% of MB and 82% of MO can be removed even in the fifth cycle, signifying the high stability and reusability efficiency of the GOPSr-2 nanocomposite.

### 3.11. Proposed Mechanism

Scheme 2 illustrates the schematic representation of the proposed adsorption mechanism of MB and MO by GOPSr-2. The adsorption mechanism can be explained as follows:

Since MB and MO are cationic and anionic in nature, electrostatic attraction is one of the major factors that enhances the efficient removal of charged dyes. GO contains hydroxyl and epoxy groups, along with an sp^2^ hybridized framework that makes it overall negatively polarized, thereby forming strong electrostatic interaction with MB for its efficient removal. On the other hand, due to the presence of a positively-charged polymeric backbone, PANI contributes to the efficient removal of MO through electrostatic attraction (this has been comprehensively discussed in Section 3.5). Furthermore, since the surface of GOPSr-2 contains hydroxyl and carboxyl functional groups on its surface, whereas MB and MO contain nitrogen atoms within their structure, the lone pair of nitrogen atoms forms intermolecular H-bonding with these functional groups, thereby aiding in their efficient removal from the aqueous solution. Moreover, MB and MO dyes are ideally planar molecules that can be easily adsorbed on the surface of GOPSr-2 via π–π interaction between the aromatic backbone of the dye and the aromatic skeleton of the GOPSr-2 adsorbent. Therefore, all these physical forces, namely, electrostatic interaction, intermolecular H-bonding, and π–π interaction, work synergistically and lead to the efficient removal of dyes from aqueous solutions within a short period of time.

## 4. Conclusions

Polyaniline-coated graphene oxide doped with SrTiO_3_ nanocube nanocomposites has been successfully synthesised via a simple in situ oxidative polymerisation technique. PANI was successfully coated onto the GO sheets and SrTiO_3_ nanocubes were successfully incorporated within the matrix of nanocomposite. The surface morphology of GO was completely transformed from aggregated sheets to more segregated ones upon incorporation of PANI and SrTiO_3_ nanocubes, as depicted by FESEM analysis. The synthesised nanocomposites exhibited greater adsorption efficiencies as compared to bare GO and PANI homopolymers, suggesting a synergistic phenomenon taking place between the polymer chain, graphene sheets, and cubic nanoparticles. The removal efficiency of MB was found to be largely unaffected by a change in pH, whereas MO was found to be pH-dependent with maximum dye adsorption at pH 7. No significant decrease in adsorption capacity was observed even at high salt concentrations. Furthermore, the synthetic methodology proposed here may be used for the synthesis of numerous SrTiO_3_ nanocube-doped GO nanocomposites materials utilising other conducting polymers, which may help address present concerns about environmental pollution.

## Supplementary Materials

The following are available online at www.mdpi.com/2073-4360/8/8/305/s1, Figure S1: FESEM images of (a) and (b) GOPSr-2 nanocomposite at different magnifications, Figure S2: TEM images GOPSr-2 nanocomposite at different magnifications, Figure S3: EDX spectrum of GOPSr-2 nanocomposite, Figure S4: FESEM image of GOPSr-2 nanocomposite after fifth reusability cycle.
