# Supplementary material for: Synthesis of Polyaniline-Coated Graphene Oxide@SrTiO3 Nanocube Nanocomposites for Enhanced Removal of Carcinogenic Dyes from Aqueous Solution
Source: Polymers (Basel). 2016 Sep 2;8(9):305. doi: 10.3390/polym8090305 (PMC6432135; doi:10.3390/polym8090305)
Supplement: Supplementary file 1 [file polymers-08-00305-s001.pdf]

Supplementary Materials: Synthesis of Polyaniline coated Graphene Oxide @ SrTiO3 Nanocube Nanocomposites for Enhanced Removal of Carcinogenic Dyes from Aqueous Solution

Syed Shahabuddin, Norazilawati Muhamad Sarih, Muhammad Afzal Kamboh, Hamid Rashidi Nodeh and Sharifah Mohamad

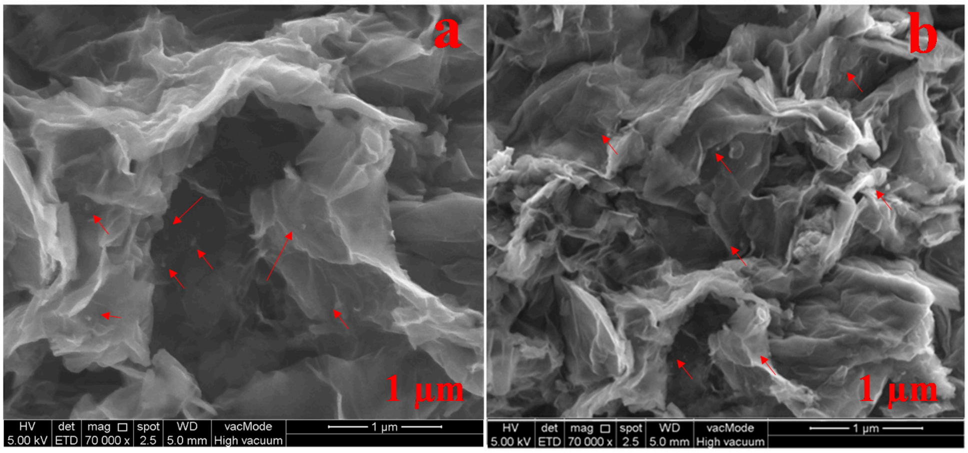

Figure S1. FESEM images of (**a**,**b**) GOPSr-2 nanocomposite at different magnifications.

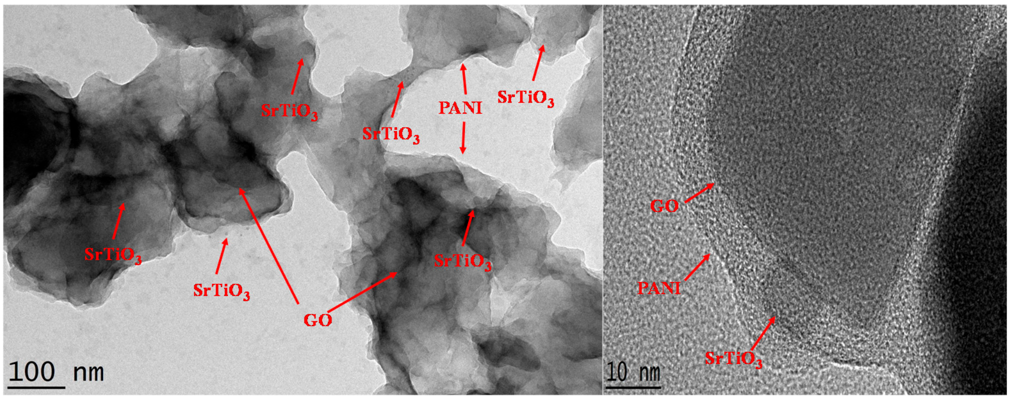

Figure S2. TEM images GOPSr-2 nanocomposite at different magnifications.

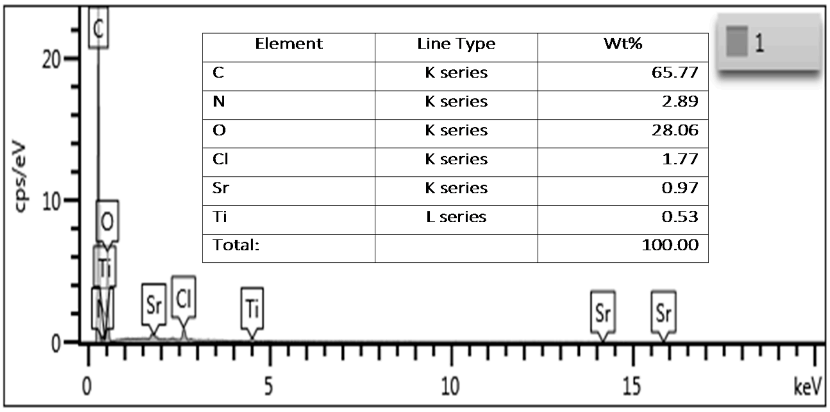

Element	Line Type	Wt%
C	K series	65.77
N	K series	2.89
O	K series	28.06
Cl	K series	1.77
Sr	K series	0.97
Ti	L series	0.53
Total:		100.00

Figure S3. EDX spectrum of GOPSr-2 nanocomposite.

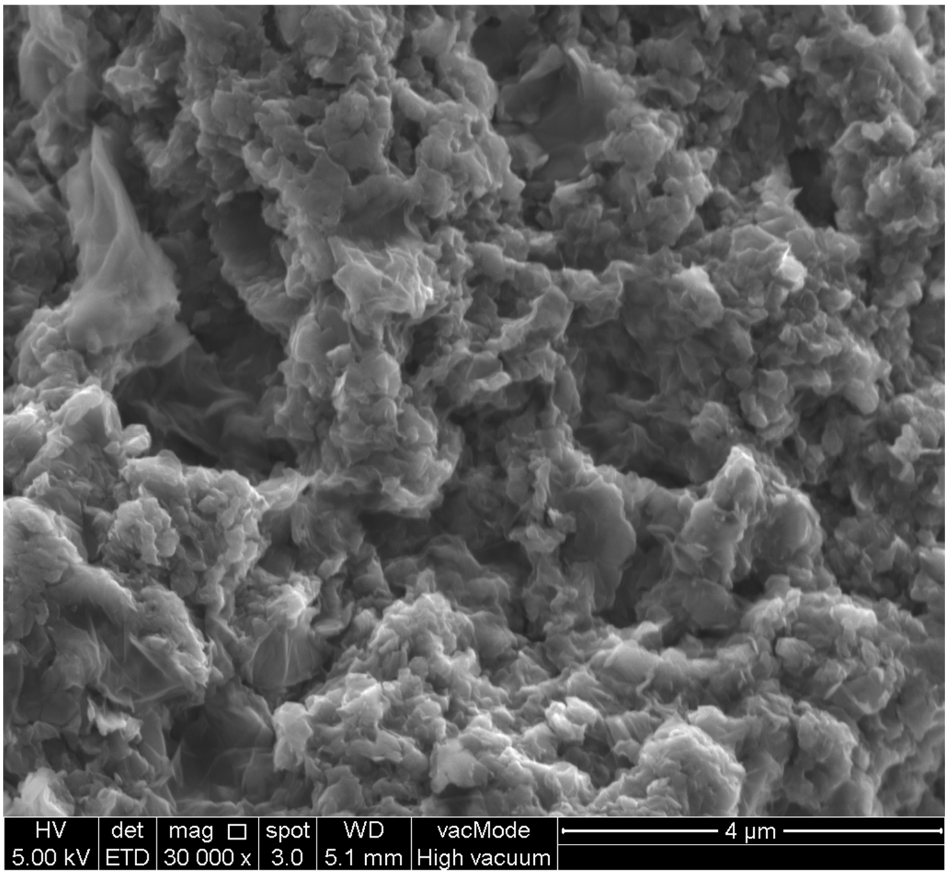

Figure S4. FESEM image of GOPSr-2 nanocomposite after fifth reusability cycle.
